# Comparisons of Three Methods for Myopia Control in Adolescents

**DOI:** 10.1155/2022/9920002

**Published:** 2022-09-29

**Authors:** Ling-fang Du, Fang He, Hua-xia Tan, Na Gao, Wei-qiong Song, Yu-xiu Luo

**Affiliations:** Optometry Center, The First People's Hospital of Chen Zhou City, Chenzhou 423000, Hunan, China

## Abstract

**Objective:**

A rising trend in electronic use has increased the prevalence of myopia in adolescents, but the optimal approach to controlling myopia remains undetermined. Here, we explored the effects of common single vision (SV) spectacle lenses combined with 0.01% atropine eye drops (SV + A), orthokeratology (OK) lenses, and peripheral defocus (PD) spectacle lenses on myopia control in adolescents.

**Methods:**

Totally 150 myopic adolescent patients (300 eyes) receiving treatment at The First People's Hospital of Chenzhou City were enrolled. According to doctors' advice and guardians' wishes, the patients were divided into SV + A group, OK group, and PD group, with each group consisting of 50 cases (100 eyes). The spherical equivalent, axial length, accommodative response index (accommodative sensitivity and accommodative lag), and intraocular pressure were compared before and after 12 months of wearing lenses, and the complications were recorded.

**Results:**

Before wearing lenses, there was no statistical significance in baseline characteristics such as age, gender, and spherical equivalent among the three groups (*P* > 0.05). After wearing lenses, the increase in spherical equivalent and axial length in the SV + A and OK groups were lower than in the PD group (*P* < 0.05), and the SV + A group had the lowest axial length growth. Compared with the SV + A group, accommodative sensitivity was much higher and accommodative lag was significantly lower in the OK and PD groups (*P* < 0.01). In addition, there was no significant difference in intraocular pressure before and after wearing lenses among the three groups (*P* > 0.05). Though the OK group patients had more complications, the difference was not statistically significant (*P* > 0.05).

**Conclusion:**

SV + A, OK, and PD lenses can effectively control the progression of myopia in adolescents, but SV + A and OK lenses exhibited more significant effects.

## 1. Introduction

Myopia, one of the most common types of refractive error, refers to the phenomenon of unclear retinal imaging. Epidemiological studies showed that it affects nearly one-third of the US population [1], but the prevalence is highest in Asian children [2–4], followed by Hispanic, and then black and white children [4, 5]. Myopia tends to develop by 8 years of age and may progress through 15 or 16 years of age [6], with an average progression rate of approximately 0.50 diopter (D) per year [7, 8].

Specifically, excessive axial elongation induces light rays from distant objects to focus in front of the retina, thereby making retinal imaging unclear [9]. In recent years, due to the popularization and increasing use of electronic products, the prevalence of myopia has been growing year by year, and the patients with myopia also tend to be younger. At present, myopia has become a widespread public health concern worldwide that seriously threatens the physical and mental health of adolescents [10]. Therefore, it is very important to strengthen the prevention and control of myopia in adolescents, and there have been some previous studies on the progress of myopia control [11, 12].

In 2018, the Chinese Ministry of Education released the Comprehensive Plan to Prevent Nearsightedness among Children and Teenagers (CPPNCT) to reduce myopia incidence and control myopic progression in China through recommendations spanning from home-based to school-based interventions, including the promotion of outdoors and physical activities, increasing sunlight exposure, decreasing screen use time, and guidance on diet and sleep [13]. Generally, approaches to prevent and treat myopia include optical correction, application of cycloplegic, hypotensive eye drops, contact lenses, visual training, and enhanced nutrition [14]. Of these, optical correction is the most commonly used for myopia control in clinics. Currently, the main myopia correction methods consist of wearing common single vision (SV) spectacle lenses, orthokeratology (OK) lenses, and peripheral defocus (PD) spectacle lenses [15]. Despite the advantages of safety, effectiveness, cheapness, convenience, and noninvasiveness, SV lenses have poor effects on myopia control due to their paracentral defocus phenomenon [16]. Therefore, atropine usually serves as supplementation of SV lenses for myopia prevention. It was reported that atropine targets biological receptors in the retina, sclera, and choroid can regulate growth factors at these sites and inhibit axial growth, thereby controlling myopia progression [17]. Several clinical trials have pointed out that low-concentration atropine eye drops could effectively control myopia progression in adolescents [18–20]. According to a recent meta-analysis, 0.01% atropine was found to be effective and safe for myopia control in adolescents [21]. Hence, SV lenses combined with 0.01% atropine eye drops are also a good method to control myopia.

OK lens is a special rigid gas permeable (RGP) lens that improves visual acuity by changing the corneal convergence morphology [22] and makes up for some defects of SV lenses. Moreover, OK lenses can effectively control the progression of myopia, improve myopic anisometropia, relieve visual discomfort, and ultimately improve binocular visual function [23, 24]. Through special optical design, PD lenses can correct central refractive error and utilize positive relative peripheral refraction to correct peripheral retinal hyperopic defocus in myopic eyes, thereby inhibiting axial growth induced by peripheral retinal hyperopic defocus [25]. Furthermore, the appearance of PD lenses is designed as a concentric circle (360 degree), the refractive power can decrease from a central to a peripheral area, and the lens frame material is soft and safe without metal parts. Thus, PD lenses satisfy the psychological and physiological needs of adolescents in China [26].

However, there is a lack of comparative studies on the effects of the above three methods on myopia in adolescents. In view of this, this study intended to investigate the effects of the above three methods on spherical equivalent, axial length changes, and accommodative response indexes in adolescents.

## 2. Subjects and Methods

### 2.1. Study Subjects

A total of 150 myopic adolescent patients (300 eyes) receiving treatment at The First People's Hospital of Chenzhou City (Chenzhou, Hunan, China) from June 2020 to May 2021 were selected as the study subjects. The study inclusion criteria were as follows: (1) age: 8-15 years; (2) refractive power: 1.00 to −5.00 D, astigmatism ≤−2.00D, and best corrected visual acuity ≥0.8; and (3) patients who could cooperate in regular hospital re-examination. The exclusion criteria included patients with OK lens contraindications and patients who needed long-term medical treatment due to immunodeficiency, psychiatric disorders, and malignant tumors.

**Figure 1:**
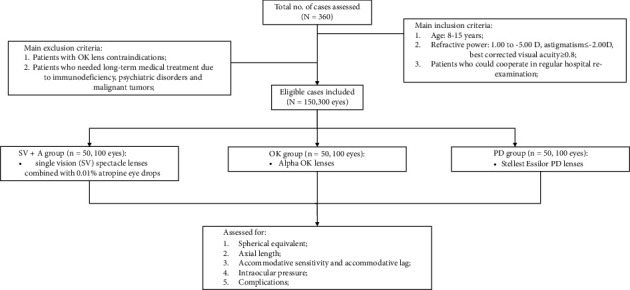
Flowchart of the study population.

Fitting methods were selected based on the doctors' advice and the guardian's wishes. Specifically, patients in the SV + A group (*n* = 50, 100 eyes) were required to wear SV lenses, in the OK group (*n* = 50, 100 eyes), they were given OK lenses to wear, and in the PD group (*n* = 50, 100 eyes) they were given PD lenses to wear. This study was approved by the Ethics Committee of The First People's Hospital of Chenzhou City (2021018H). This study was performed in strict accordance with the Declaration of Helsinki, and the guardians of the patients were aware of the purpose and precautions of this study and volunteered to sign an informed consent form.  Figure 1 shows the flow chart of the participants.

### 2.2. Interventions

In the SV + A group, patients were asked to wear the commonly available SV lenses (Zeiss, Oberkochen, Germany) on the market according to the outcomes of refraction and trial of the lens and were also given 0.01% atropine eye drops (Shenyang Xiangqi Pharmaceutical Co, Ltd). In the OK group, after routine ocular examination, the patients were required to wear Alpha OK lenses (Alpha, Nagoya, Japan), and standard lenses were adjusted according to the specific situations; after determining the lens parameters, the most appropriate OK lenses were selected, and the patients were instructed to wear them for 8-10 hours daily. In the PD group, the patients underwent eye position and accommodative function examination, followed by refraction and trial lenses, and finally, they were asked to wear PD lenses (Essilor, Paris, France). Spherical equivalent, axial length, accommodative sensitivity, accommodative lag, and intraocular pressure were examined before wearing lenses in all three groups. Complications needed to be observed during lens wearing. After 12 months of wearing the lenses, all of the above indicators were re-examined. If there were any eye discomfort symptoms, another re-examination was required to be performed in time.

### 2.3. Outcome Measures

#### 2.3.1. Spherical Equivalent

Spherical equivalent served as one of the criteria for screening and diagnosing myopia. The patients received cycloplegic refraction first, followed by manual skiascopy and KR-800 Auto Kerato-Refractometer (Topcon, Tokyo, Japan) measurement. Next, the best corrected visual acuity was obtained, and spherical equivalent was calculated according to the formula of “spherical equivalent = sphere (D) + 1/2 cylinder (D).”

#### 2.3.2. Axial Length

Axial length was measured using an IOLMaster biometer (Zeiss, Oberkochen, Germany). The axial length of normal adults was about 24 mm, which varied by 1 mm according to refractive calculations, indicating about 3 d of changes in refractive power.

#### 2.3.3. Accommodative Sensitivity and Accommodative Lag

As an evaluation index of visual function, accommodative sensitivity was the speed of accommodation response made by the human eye to different accommodation stimuli. Briefly, accommodative sensitivity was an index to evaluate whether the eye could change the accommodation smoothly and effectively. In this paper, the accommodative flippers were applied to measure accommodative sensitivity. Accommodative lag referred to the amount of accommodative response of the eye less than the amount of accommodative stimulation. Generally, accommodative lag was an indicator to evaluate the advantages and disadvantages of accommodative function. A phoropter was utilized to detect the accommodative lag.

#### 2.3.4. Intraocular Pressure

NIDEK NT-530 Non-Contact Tonometer (NIDEK, Tokyo, Japan) was adopted to evaluate intraocular pressure.

#### 2.3.5. Complications

Keratitis, corneal infection, foreign body sensation, and visual abnormality were recorded.

### 2.4. Statistical Analysis

The data were analyzed using the Statistical Package For the Social Sciences (SPSS; IBM, Armonk, USA) software version 22.0, enumeration data were presented as *n* (%), and comparisons were checked using the *χ*^2^ test or Fisher's exact test. Measurement data were expressed as mean ± standard deviation (SD), differences between groups were compared using the one-way variance test, and further pairwise comparisons were performed using the least significant difference (LSD) *t*-test. Additionally, paired *t*-test was used for comparison before and after wearing lenses. *P* < 0.05 was considered statistically significant.

## 3. Results

### 3.1. Baseline Characteristics

Each group contained 50 adolescent patients (100 eyes). Specifically, the SV + A group included 25 males and 25 females (age: 8-14 years), the OK group included 26 males and 24 females (age: 8-15 years), and the PD group included 28 males and 22 females (age: 9-15 years). There were no significant differences in general data such as age, gender, spherical equivalent, and axial length among the three groups (*P* > 0.05) (Table 1).

### 3.2. Comparison of the Change of Spherical Equivalent before and after Wearing Lenses between the Three Groups

Before wearing lenses, there was no significant difference in spherical equivalent among the three groups (*P* > 0.05). After wearing lenses, the spherical equivalent and its increase degree were significantly higher in the PD group than in the SV + A and OK groups (*P* < 0.01), while the differences between the SV + A and OK groups were not significant (Table 2). Briefly, the SV + A group and the OK group exhibited more significant myopia control effects than the PD group.

### 3.3. Comparison of Axial Length Changes before and after Wearing Lenses between the Three Groups

There were no obvious differences in axial length among the three groups before and after wearing lenses (*P* > 0.05), while the increase of axial length in the SV + A group was significantly lower than that in the OK group and PD group (*P* < 0.01), and the increase of axial length in the OK group was much lower than in the PD group (*P* < 0.01) (Table 3).

### 3.4. Comparison of Accommodation Response and Intraocular Pressure before and after Wearing Lenses between the Three Groups

Before wearing lenses, there was no significant difference in accommodative sensitivity, accommodative lag, and intraocular pressure among the three groups (*P* > 0.05). After wearing glasses, the OK group and PD group showed much higher accommodative sensitivity and significantly lower accommodative lag compared with the SV + A group (*P* < 0.001). In contrast, the difference between the OK group and PD group was not statistically significant (*P* > 0.05) (Table 4). In addition, there was no significant difference in intraocular pressure after wearing lenses among the three groups (*P* > 0.05), indicating that the three myopia correction methods had no significant side effects on intraocular pressure in adolescents.

### 3.5. Comparison of Complications after Wearing Lenses between the Three Groups

The main complications after wearing lenses included keratitis, corneal infection, foreign body sensation, and visual abnormalities, among which visual abnormalities were the major complications in all three groups. There were a few foreign body sensation complications in the SV + A group and PD group, while OK group patients suffered from more complications and were prone to develop keratitis, corneal infection, foreign body sensation, and other discomforts (Table 5).

## 4. Discussion

The mechanism of myopia was not fully understood, and most scholars believe that myopia is related to visual environment, behavior, and genetics [27]. Behavioral adjustment is considered the main cause of myopia in adolescents in the growth and development stage [28]. Myopia leads to asthenopia, reduces distance vision, and affects adolescents' learning and life [29]. Currently, myopia in adolescents cannot be completely cured. Generally, myopia in adolescents is corrected by nonsurgical modalities such as wearing spectacle lenses, OK lenses, and PD lenses [15].

There is a paracentral defocus phenomenon in wearing SV lenses, but fortunately 0.01% atropine eye drops not only have few adverse reactions and a low rebound rate but can also be used as a supplementation for SV glasses. A long-term application of 0.01% atropine eye drops combined with SV lenses has shown good effects on myopia control [30]. Due to the special design of inverse geometry, OK lenses can flatten the central corneal region, then the central axis can be imaged in the macular area, and the periphery can be imaged on the retina. The above procedure allows OK lenses to eliminate the paracentral hyperopic defocus phenomenon and control myopia progression. Yuan et al. [31] reported that wearing OK lenses was a safe method to correct myopia. Indeed, the short-term wearing of OK lenses could effectively improve uncorrected visual acuity in myopic adolescent patients without affecting their central corneal thickness and corneal endothelial cells. PD lenses, emerging optical correction devices in 2013 in China, can effectively reduce paracentral hyperopic defocus. Compared with OK lenses, PD lenses are suitable for a wider group of patients [32]. The above three correction methods were compared in this study and the results were shown as follows. After wearing lenses, the spherical equivalent and axial length of each group of patients increased to a lesser extent than before wearing lenses, indicating that the three myopia correction methods could effectively control the progression of myopia. Among the three groups, the SV + A and OK groups had better effects on myopia control than the PD group. In addition, the increase in axial length in the SV + A group was significantly lower than in the OK and PD groups. Overall, common single vision spectacle lenses combined with the use of 0.01% of atropine eye drops showed promising efficacy in potentially inhibiting axial growth [33, 34].

An abnormal accommodative function is also one of the important causes of myopia in adolescents. Specifically, accommodative sensitivity and accommodative lag are two key factors associated with the development of myopia [35]. Prolonged close eye use can make accommodation inaccurate, increase accommodation lag, reduce accommodation sensitivity, aggravate hyperopic defocus, increase axial length, and ultimately promote myopia progression [36, 37]. In this study, after wearing lenses, the accommodation sensitivity of the OK group and PD group was significantly higher, while the accommodation lag was significantly lower than that of the SV + A group. The above findings suggested that wearing OK lenses and wearing PD lenses could improve the myopia accommodation response in adolescents, thereby improving their visual function and slowing down the growth of myopia. Patients in the SV + A group showed a mild decrease in accommodative function, but it did not affect normal learning and life. Besides, there was no significant difference between the three groups in intraocular pressure and total incidence of complications after wearing lenses.

There were some advantages and disadvantages to the three myopia correction methods used in this study. Although SV + A was effective and convenient in controlling myopia by inhibiting axial growth, its effects on regulating visual function were not as good as OK lenses and PD lenses, and some patients suffer photophobia, blurred vision, or drug allergy [38]. The effects of OK lenses in controlling myopia and regulating visual function were significant, which was consistent with previous studies [39], while OK lenses were not suitable for patients with myopia higher than −6.00 D and were accompanied by a certain risk of complications. Wearing PD lenses was not as effective as wearing SV + A and OK lenses in controlling myopia, but the effects of PD lenses on regulating visual function were better than SV + A, and its correction degree reached −12.00D. Moreover, compared with OK lenses, PD lenses are not only more convenient and safe but also more easily accepted by adolescents and their guardians [32]. All in all, the three correction methods in this paper have their own advantages and disadvantages and need to be selected according to the actual situation.

The number of contact lens wearers has steadily increased over the past decades. Convenience, efficiency, and availability of different types of lenses have made them popular among young people [40]. However, the rise in the use of contact lenses has increased the number of people at risk for contact lens-related complications. The main reasons for patient dissatisfaction after wearing lenses observed in this study were keratitis, corneal infection, foreign body sensation, and visual abnormalities. Visual abnormalities such as residual ametropia represent one of the most common reasons for patient dissatisfaction related to increased sensitivity to residual refractive error. Management based on the severity might include changing to other types of lenses or surgery [41]. A common cause of infection might be the use of water instead of sterile cleaning solutions that can warp lenses, as well as function as a means to introduce pathogens to lenses. In addition, over time, protein and lipid deposits form on the surfaces of contact lenses [42]. If left untreated, they can irritate the eye. Microbes can form films over the lens, impairing vision and putting the eye at risk of infection. It should also be noted that if contacts are not removed for sleeping, the eye can suffer damage due to poor airflow [43]. Further, irritants and microbial action can lead to serious complications, including ulcers and permanent vision loss due to infection. Thus, for lenses to work properly, they must be cleaned and maintained according to doctor's guidelines. It is very important to have good contact lens hygiene. This includes not sleeping, showering, or swimming while wearing contacts to reduce the risk of serious complications. In addition, the risk of infectious keratitis can be reduced by using daily disposable contacts [43]. However, a relatively safe and reliable alternative to contacts is Lasik laser eye surgery, which has been shown to improve vision and remove the need for corrective devices.

There were some potential limitations in this study. First, the sample size collected in this study was small, the follow-up time was short, and indicators such as tear film changes were not analyzed. Second, we did not analyze the effects of eye use time on the results and did not record the frequency and mode of patients wearing lenses. Lastly, wearing lenses without the doctor's advice may also affect the effects of myopia control to a certain extent. Thus, prospective studies using larger sample size and better-designed comparative groups are required to confirm the optimal approach for myopia control in adolescents.

## 5. Conclusion

In summary, our results showed that all three correction methods investigated in this study effectively controlled myopia progression in adolescents, but the SV + A and OK lenses exhibit more significant effects in myopia control. However, each correction method has its underlying advantages and disadvantages, and patients and their guardians should choose the most appropriate one based on their needs. In addition, many of the complications observed might be preventable, indicating greater awareness of good contact lens hygiene among adolescents.

## Figures and Tables

**Table 1:** Comparisons of the baseline characteristics of the included patients.

Variable	SV + A (*n* = 50/100)	OK (*n* = 50/100)	PD (*n* = 50/100)	*χ* ^2^ * /F*	*P*
Age (years)	11.96 ± 2.33	11.92 ± 2.46	11.97 ± 2.32	0.012	0.988
Gender (male/female)	25/25	26/24	28/22	0.374	0.829
Spherical equivalent (D)	−2.90 ± 1.42	−2.87 ± 1.41	−2.86 ± 1.38	0.022	0.978
Axial length (mm)	24.11 ± 0.78	24.17 ± 0.84	24.15 ± 0.81	0.142	0.868
Accommodative sensitivity (CPM)	7.81 ± 1.04	7.83 ± 1.05	7.84 ± 1.08	0.021	0.979
Accommodative lag (D)	1.02 ± 0.17	1.03 ± 0.20	1.00 ± 0.19	0.667	0.514
Intraocular pressure (mm·Hg)	16.75 ± 2.25	16.64 ± 2.27	16.81 ± 2.30	0.144	0.866

SV + A: single vision spectacle lenses combined with 0.01% atropine eye drops; OK: orthokeratology lenses; PD: peripheral defocus spectacle lenses.

**Table 2:** Comparison of spherical equivalent before and after wearing lenses between the SV + A, OK, and PD groups.

Factor	Eyes	Before wearing lenses (D)	One year after wearing lenses (D)	Difference
SV + A	100	−2.90 ± 1.42	−3.15 ± 0.87	−0.25 ± 0.21
OK	100	−2.87 ± 1.41	−3.26 ± 0.78	−0.39 ± 0.24
PD	100	−2.86 ± 1.38	−3.52 ± 0.93	−0.56 ± 0.25
*F*		0.022	4.856	44.032
*P*		0.978	0.008	<0.001

SV + A: single vision spectacle lenses combined with 0.01% atropine eye drops; OK: orthokeratology lenses; PD: peripheral defocus spectacle lenses.

**Table 3:** Comparison of axial length changes before and after wearing lenses between the SV + A, OK, and PD groups.

Factor	Eyes	Before wearing lenses (mm)	One year after wearing lenses (mm)	Difference
SV + *A*	100	24.11 ± 0.78	24.28 ± 0.76	0.17 ± 0.05
OK	100	24.17 ± 0.84	24.36 ± 0.83	0.19 ± 0.03^*∗∗*^##
PD	100	24.15 ± 0.81	24.41 ± 0.79	0.26 ± 0.02^*∗∗*^
*F*		0.142	0.682	176.316
*P*		0.868	0.506	<0.001

SV + A: single vision spectacle lenses combined with 0.01% atropine eye drops; OK: orthokeratology lenses; PD: peripheral defocus spectacle lenses. ^*∗∗*^*P* < 0.01*vs*. SV + A group. ^##^*P* < 0.01*vs*. PD group.

**Table 4:** Comparison of accommodation response before and after wearing lenses between the SV + A, OK, and PD groups.

Factor	Eyes	Accommodative sensitivity (CPM)		Accommodative lag (D)		Intraocular pressure (mm·Hg)	
		Before wearing lenses	After wearing lenses	Before wearing lenses	After wearing lenses	Before wearing lenses	After wearing lenses
SV + A	100	7.81 ± 1.04	7.95 ± 1.23	1.02 ± 0.17	0.98 ± 0.15	16.75 ± 2.25	16.92 ± 2.33
OK	100	7.83 ± 1.05	9.34 ± 1.22^*∗∗∗*^	1.03 ± 0.20	0.78 ± 0.12^*∗∗∗*^	16.64 ± 2.27	16.76 ± 2.31
PD	100	7.84 ± 1.08	9.29 ± 1.27^*∗∗∗*^	1.00 ± 0.19	0.80 ± 0.11^*∗∗∗*^	16.81 ± 2.30	16.93 ± 2.38
*F*		0.021	40.420	0.667	74.286	0.144	0.166
*P*		0.979	<0.001	0.514	<0.001	0.866	0.847

SV + *A*: single vision spectacle lenses combined with 0.01% atropine eye drops; OK: orthokeratology lenses; PD: peripheral defocus spectacle lenses. ^*∗∗∗*^*P* < 0.001*vs*. SV + A group.

**Table 5:** Comparison of complications between the SV + A, OK, and PD groups.

Factor	Eyes	Keratitis (%)	Corneal infection (%)	Foreign body sensation (%)	Visual abnormalities (%)	Total incidence (%)
SV + A	100	0 (0.00)	0 (0.00)	2 (2.00)	10 (10.00)	12 (12.00)
OK	100	4 (4.00)	3 (3.00)	3 (3.00)	9 (9.00)	19 (19.00)
PD	100	0 (0.00)	0 (0.00)	1 (1.0)	10 (10.00)	11 (11.00)
*χ* ^2^		—	—	—	—	2.335
*P*		0.020	0.053	0.613	0.969	0.311

SV + A: single vision spectacle lenses combined with 0.01% atropine eye drops; OK: orthokeratology lenses; PD: peripheral defocus spectacle lenses.

## Data Availability

The data used to support the findings of this study are available from the corresponding author upon request.
